# A new prognostic model including immune biomarkers, genomic proliferation tumor markers (*AURKA* and *MYBL2*) and clinical-pathological features optimizes prognosis in neoadjuvant breast cancer patients

**DOI:** 10.3389/fonc.2023.1182725

**Published:** 2023-05-29

**Authors:** Esmeralda García-Torralba, Esther Navarro Manzano, Gines Luengo-Gil, Pilar De la Morena Barrio, Asunción Chaves Benito, Miguel Pérez-Ramos, Beatriz Álvarez-Abril, Alejandra Ivars Rubio, Elisa García-Garre, Francisco Ayala de la Peña, Elena García-Martínez

**Affiliations:** ^1^ Department of Haematology and Medical Oncology, University Hospital Morales Meseguer, Murcia, Spain; ^2^ Department of Medicine, Medical School, University of Murcia, Murcia, Spain; ^3^ Instituto Murciano de Investigación Biosanitaria (IMIB), Murcia, Spain; ^4^ Department of Pathology, University Hospital Morales Meseguer, Murcia, Spain; ^5^ Medical School, Catholic University of Murcia, Murcia, Spain

**Keywords:** Breast cancer, neoadjuvant chemotherapy, neutrophil-to-lymphocyte ratio, tumor-infiltrating lymphocytes, proliferation markers

## Abstract

**Background:**

Up to 30% of breast cancer (BC) patients treated with neoadjuvant chemotherapy (NCT) will relapse. Our objective was to analyze the predictive capacity of several markers associated with immune response and cell proliferation combined with clinical parameters.

**Methods:**

This was a single-center, retrospective cohort study of BC patients treated with NCT (2001-2010), in whom pretreatment biomarkers were analyzed: neutrophil-to-lymphocyte ratio (NLR) in peripheral blood, CD3+ tumor-infiltrating lymphocytes (TILs), and gene expression of AURKA, MYBL2 and MKI67 using qRT-PCR.

**Results:**

A total of 121 patients were included. Median followup was 12 years. In a univariate analysis, NLR, TILs, AURKA, and MYBL2 showed prognostic value for overall survival. In multivariate analyses, including hormone receptor, HER2 status, and response to NCT, NLR (HR 1.23, 95% CI 1.01-1.75), TILs (HR 0.84, 95% CI 0.73-0.93), AURKA (HR 1.05, 95% CI 1.00-1.11) and MYBL2 (HR 1.19, 95% CI 1.05-1.35) remained as independent predictor variables.

**Conclusion:**

Consecutive addition of these biomarkers to a regression model progressively increased its discriminatory capacity for survival. Should independent cohort studies validate these findings, management of early BC patients may well be changed.

## 1 Introduction

Neoadjuvant chemotherapy (NCT) is the first treatment option for locally advanced and inflammatory breast cancer (BC). It is also a standard treatment in HER2-positive (HER2+) and triple-negative (TNBC) early BC (1). NCT increases the rate of conservative surgery, enables treatment response monitoring and the selection of adjuvant treatment according to risk, and provides unique opportunities for developing novel and individualized therapeutic strategies (1, 2).

Risk stratification is critical in BC patients receiving NCT, as residual pathological disease can affect postoperative decision-making (3). Pathological complete response (pCR) has been classically considered a robust predictor of recurrence, disease-free survival (DFS), and overall survival (OS), especially in the context of the most aggressive subtypes, i. e., HER2+BC and TNBC (2, 4, 5). Despite curative intent, up to 30%-40% of these cases, including some who achieved pCR, will relapse in the first 5 years of follow-up (6). It is crucial that we identify other prognostic factors that can help distinguish individuals who will relapse despite having achieved pCR after NCT.

The adaptive and the innate immune response play a pivotal role in tumor immunosurveillance and can limit tumor development and growth, and determine response to new therapeutic approaches, such as immunotherapy. The role of the immune response in BC has not been fully elucidated. Nevertheless, there is growing evidence that the tumor immune microenvironment plays a key role in the response to different cancer treatments and in prognosis (7).

Several immunological parameters are being investigated or developed as intermediate biomarkers of cancer regression, progression, or recurrence. The most extensively studied marker to date is probably tumor lymphocytic infiltration (8). Tumor-infiltrating lymphocytes (TILs) have been proposed as a predictor of response and prognosis in HER2+ BC and TNBC subtypes, but their involvement in luminal BC is less clear (7). The characterization of the immune cell population in BC and its activation status is mandatory to improve prognosis and to predict treatment response (9).

The chronic inflammatory response is also closely linked to the development and prognosis of certain cancers (10). There is mounting evidence that the total leukocyte count and, most notably, the neutrophil-to-lymphocyte ratio (NLR) prior to embarking on anticancer treatment predict an adverse clinical outcome in several solid tumor (11, 12). This marker is particularly interesting since it integrates the subject’s immune response capacity with their inflammatory status, which tends to correlate with tumor progression and poor prognosis (13). In BC patients, an elevated NLR has been implicated in decreased survival, above all in localized stages (14–16).

The expression of proliferation-related genes in BC is linked to more aggressive subtypes (luminal B and non-luminal subtypes) and forms the foundation for the recent inclusion of clinical KI67 determinations as a predictive and prognostic immunohistochemical (IHC) marker (17). Furthermore, proliferation gene expression is key to understanding biological diversity in luminal BC (18). In fact, gene testing to determine the risk of recurrence includes this information (18–20).

The aurora A kinase (*AURKA*) gene codifies a serine/threonine kinase with a key role in mitosis regulation that works as an oncogene promoting tumorigenesis (21, 22). Several signaling pathways have been related with *AURKA*, including PI3K-Akt, Wnt, Hippo, p53 and FOXO (21). Additionally, *AURKA* is a proliferation marker and its overexpression has also been linked to unfavorable prognosis in BC (23, 24). *AURKA* has been proposed as a possible biomarker and therapeutic target in chemotherapy and hormonotherapy resistance (25–31).

*MYBL2* overexpression has been described as a robust marker of replicative instability (RIN) that can occur in multiple tumor and is a driver in progressive disease and treatment resistance (32). In BC patients, a high *MYBL2* proto-oncogene level may also be a biomarker of adverse prognosis (33, 34) and could promote tumor invasion by the induction of epithelial–mesenchymal transition (EMT) and modulation of immune microenvironment (35, 36). *In vitro* studies have demonstrated its role in tamoxifen resistance (37). Interestingly, Guarneri et al. in the ShortHER phase III trial found that HER2-enriched BC tumor with mutated *PIK3CA* had an upregulated expression of *MKI67*, *MYBL2*, *ESR1*, *PDCD1* and other genes, but only *MYBL2* and *PDCD1* were related with a better DFS (38).

A single perfect biomarker is unlikely to exist. Recent publications suggest that combined biomarker determination can potentially yield more complete and relevant information (39). Importantly, the predictive value of proliferative signatures and immune signatures are apparently independent, at least in TNBC (40). Combining two proliferation markers such as *AURKA* and *MYBL2*, with different biological meaning and with diverse impact on therapeutic resistance, might also improve prognostic stratification. Hence, given the need to enhance currently available prognostic systems and to optimize therapeutic strategies, we have analyzed the prognostic contribution of several immune response-related markers, both in the tumor microenvironment and systemically, in combination with tumor proliferation and clinical-pathological features, in BC patients treated with NCT.

## 2 Materials and methods

### 2.1 Study cohort and clinical management

A series of patients consecutively diagnosed with stage II or III BC who received NCT in the Hematology and Medical Oncology Department of a single tertiary hospital (University Hospital Morales Meseguer, Murcia, Spain) between July 2001 and October 2010 was retrospectively analyzed. Clinical evaluation at diagnosis was conducted as per clinical practice criteria, including breast MRI and axillary ultrasound in all cases. Pre-NCT lymph node status was determined by ultrasound-guided fine needle aspiration or sentinel node biopsy. Treatment followed local protocols, in accordance with international recommendations applicable at the time of diagnosis (41). NCT included taxanes and anthracyclines, as well as trastuzumab in patients with HER2+ tumor. After surgery, hormone therapy was prescribed to all patients with hormone receptor (HR)-positive tumor and adjuvant trastuzumab in HER2+ tumor. Adjuvant radiotherapy was administered to all patients treated with conservative surgery, and to patients undergoing mastectomy with a high risk of relapse.

Written informed consent was obtained from all patients included in the study. The study was approved by the Clinical Research and Trials Committee of the University Hospital Morales Meseguer (Internal code: EST07/15) and was conducted in accordance with the ethical principles of the Declaration of Helsinki.

### 2.2 Clinical and laboratory variables and neutrophil-to-lymphocyte ratio estimation

Demographic and clinical-pathological variables and treatment and response data were obtained from participants’ clinical records. Primary outcome variables included DFS, measured from the date of diagnosis to last follow-up or disease relapse, and OS, quantified as the date of diagnosis to the date of last follow-up or demise.

Routine laboratory parameters were collected from laboratory databases, using the closest blood count prior to the date NCT was initiated (maximum time: four weeks). Pretreatment NLR was calculated by dividing the absolute neutrophil count by the absolute lymphocyte count.

### 2.3 Histopathological evaluation of the tumor

Estrogen receptor (ER) and progesterone receptor (PR) status were assessed by IHC. Cases were considered negative when the percentage of immunoreactive tumor cells was <1%; the remaining cases (≥1% of stained tumor cells) were classified as positive. A validated IHC method (Herceptest, Dako North America, CA, USA) or fluorescent *in situ* hybridization (FISH) was used to determine HER2 status. Cases were positive if the Herceptest result was 3+ and/or FISH exhibited a HER2/CEP17 ratio ≥ 2; all others were coded as negative. pCR was defined as the absence of invasive carcinoma in the breast and axilla, regardless of the presence of carcinoma *in situ* (ypT0/Tis ypN0).

### 2.4 Tumor-infiltrating lymphocyte counts

CD3-positive TILs (TIL-CD3+) were quantified using IHC, as previously reported (9). In brief, after a pathologist (ACB) had selected tumor-predominant areas, a tissue microarray was constructed from 2 mm biopsies prior to initiating NCT treatment. Appropriate controls were included in each array. Sections measuring 4 μm were cut from the tissue microarray, deparaffinized, rehydrated, and processed by standard methods using an automated stainer (Autostainer Link 48, Dako, CA, USA). Secondary antibodies and visualization were performed using standard Dako Envision systems. All slides were simultaneously stained to avoid intersection variability. Positivity for human CD3 was tested using the polyclonal antibody IS503 (Dako, CA, USA).

After two independent observers had verified that staining was correct, each slide was scanned and digitized using an automated scanning system (Leica SCN400F). Digital images of pre-NCT samples were obtained for each tissue core and, after area quantification, adjusted morphometric analysis of the tumor area was performed with ImageJ software (National Institutes of Health, NIH, USA), including both stromal and intratumoral CD3+ cells. The results are expressed as TIL count/mm^2^.

### 2.5 RNA purification and tumor gene expression assay

Total RNA from formalin-fixed paraffin-embedded (FFPE) biopsies was extracted using a RNeasy FFPE kit (QIAGEN, MD, USA) following the supplier’s instructions. Total RNA from cells was extracted using RNAzol reagent (MRC Inc, OH, USA) and a Direct-zol RNA MiniPrep kit (ZYMO Research, CA, USA).

mRNA (with preamplification) was retrotranscribed and amplified using TaqMan^®^ Gene Expression Assays (Applied Biosystems, CA, USA) on a LightCycler^®^ 480 real-time PCR (qRT-PCR) system (Roche Diagnostics, Switzerland). Relative expression levels of each gene were calculated and quantified by 2-ΔΔCt using ACTB as the endogenous control.

### 2.6 Statistical analysis

Descriptive analyses of qualitative variables included proportions. Shapiro-Wilk tests were used to test continuous variables for normality. Continuous variables with normal distribution were presented as means ± standard deviations (SD), whereas non-normally distributed variables were reported as median and interquartile ranges (IQR). Pearson’s χ2 test was used to compare proportions or ordinal variables. Differences in means were studied with the Student’s t-test (parametric) or the Mann-Whitney U test (non-parametric).

Survival analyses of DFS and OS outcome variables were calculated using the Kaplan-Meier method and log-rank tests. The predictive impact of the different clinical, biological, and genomic variables on the outcome variables was ascertained by uni- and multivariate Cox proportional hazards regression analyses. To overcome the possibility of collinearity, variables with strong (|r| ≥ 0.5) or significant (p < 0.05) correlations were excluded. The predictive capacity of the regression models was appraised using the Akaike Information Criterion (AIC) index and Likelihood Ratio Test (LLRT). Biological and genomic variables were analyzed as continuous quantitative variables, although logarithmic or square root transformations of the TILs, *AURKA*, *MYBL2*, and *MKI67* variables were performed so that the data would comply more closely with the assumptions of the statistical procedures to be applied or to enhance interpretability. Assessment of mean calibration at 10 years was calculated for each model as observed/expected (O/E) survival ratio.

Statistical analyses were performed with STATA v.16 (StataCorp LLC, TX, USA); R version 4.2.3 and RStudio (version 2023.03.0) was also used for model assessment.

## 3 Results

### 3.1 Patients

A total of 121 NCT-treated BC patients were analyzed. Patients’ baseline characteristics are shown in **Table 1**. Median age was 56 years and most tumors were diagnosed at stage IIB or IIIA-C. Infiltrating ductal carcinoma was the most common histological type and more than half of the tumors were poorly differentiated. ICH subtype distribution revealed 63.6% HR+ (13.2% HER2+), 10.7% HER2+/HR- tumors, and 21.5% TNBC.

**Table 1:** Patients’ baseline characteristics.

Baseline characteristics	N = 121 (%)^a^	N = 47 (%)^b^	p*
Age (median, range)	56 (21-79) years	57 (21-73) years	0.711
Hormonal status			
Premenopausal Postmenopausal	60 (49.6%) 61 (50.4%)	21 (44.7%) 26 (55.3%)	0.32
Clinical stage			
IIA IIB IIIA IIIB IIIC	19 (15.7%) 34 (28.1%) 40 (33.1%) 8 (6.6%) 20 (16.5%)	10 (21.3%) 14 (29.8%) 10 (21.3%) 2 (4.3%) 11 (24.3%)	0.11
Pathological subtype			
Invasive ductal carcinoma Invasive lobular carcinoma Other	113 (93.4%) 5 (4.1%) 3 (2.5%)	44 (93.6%) 2 (4.3%) 1 (2.1%)	0.98
Histological grade			
Grade I Grade II Grade III Not reported	7 (5.8%) 39 (32.2%) 61 (50.4%) 14 (11.5%)	3 (6.5%) 19 (41.3%) 22 (47.8%) 2 (4.3%)	0.66
IHC subtype			
HR+/HER2- HR+/HER2+ HR-/HER2+ TNBC N/A	61 (50.4%) 16 (13.2%) 13 (10.7%) 26 (21.5%) 5 (4.1%)	24 (51.1%) 7 (14.9%) 9 (19.1%) 7 (14.9%) 0 (0%)	0.09
Clinical response			
CR/PR SD/PD N/A	102 (84.3%) 14 (11.6%) 5 (4.1%)	43 (91.5%) 3 (6.4%) 1 (2.1%)	0.16
Breast surgery			
Mastectomy Conservative surgery	67 (55.3%) 53 (43.8%)	22 (46.8%) 25 (53.2%)	0.37
Nodal surgery			
SLN biopsy ALND	17 (14.0%) 104 (86.0%)	10 (21.3%) 37 (78.7%)	0.06
Radiotherapy			
Pre-NCT Post-NCT	0 (0%) 106 (87.6%)	0 (0%) 41(87.2%)	0.44
Follow-up (median, range) 10-year DFS (95% CI) 10-year OS (95% CI)	12.3 years (11.82-12.79) 73.3% (64.49-80.99) 75.83% (67.17-83.18)	11.6 years (11.30-11.93) 78.73% (64.34-89.30) 80.9% (66.74-90.85)	0.66 0.59 0.64

a Total number of patients included, N = 121.

b Nested cohort for the multivariate analysis, N = 47.

ALND, axillary lymph node dissection; CR, complete response; DFS, disease-free survival; HER2, human epidermal growth factor receptor 2; HR, hormone receptor; IHC, immunohistochemical; N/A, not available; NCT, neoadjuvant chemotherapy; OS, overall survival; PD, progression disease; PR, partial response; SD, stable disease; SLN, sentinel lymph node; TNBC, triple-negative breast cancer.

*The p value refers to the result of the comparison between proportions or means of both groups.

Neoadjuvant treatment consisted primarily of sequential Adriamycin-cyclophosphamide for four cycles, followed by docetaxel for four cycles (NSABP-B27 scheme, 80.2%). The pCR rate was 17% (primary tumor pCR: 20.7%; axillary pCR: 36.4%). Median follow-up was 12.3 years, the 10-year DFS rate was 73.33%, and 10-year OS was 75.83%.

Of the total 121 participants, the full analysis of clinical variables (HR/HER2 status, response to NCT), immunological variables (NLR, TILs), and genomic markers (*AURKA*, *MYBL2*, and *MKI67*) were available for multivariate analysis in 47 patients (**Supplementary Figure 1**). Since the clinical and pathological characteristics of both groups were comparable and no statistically significant differences were found, this subgroup was representative of the total sample (**Table 1**).

### 3.2 Prognostic significance of clinical, biological, and genomic factors

We evaluated the prognostic significance of the clinical factors and the various biomarkers selected by Cox regression. NLR, TILs, and genomic markers were analyzed as continuous quantitative variables. Correlation analysis demonstrated association between genomic markers *MKI67*, and *MYBL2* (**Supplementary Table 1**). Notably, there was no correlation between pretreatment NLR and TILs in the pretreatment biopsy (|r|= 0.087, p = 0.503).

**Figure 1:**
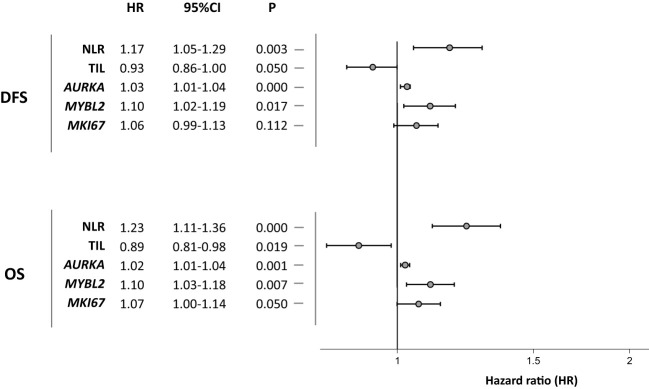
Univariate associations between immune response-related and proliferation markers and disease-free survival and overall survival.

In a univariate analysis (**Figure 1**), pCR (N = 119) was a protective factor for DFS (HR 0.13, 95% CI 0.02-0.94) and OS (HR 0.30, 95% CI 0.07-1.27), albeit falling short of statistical significance. Results were similar for patients with HR+ and HER2+, in terms of DFS (HR 0.91, 95% CI 0.46-1.78 and HR 1.15, 95% CI 0.56-2.38, respectively) and OS (HR 0.97, 95% CI 0.46-2.06 and HR 0.76, 95% CI 0.31-1.85, respectively).

The NLR biomarker in peripheral blood (N = 101) was found to have prognostic implications. Increased NLR was associated with lower DFS (HR 1.17, 95% CI 1.05-1.29) and OS (HR 1.23, 95% CI 1.11-1.36). At the tumor level, the immunological marker TIL (N = 71) exhibited a statistically significant association in the opposite direction from NLR. Patients with higher tumor lymphocytic infiltration had a better prognosis in terms of DFS (HR 0.93, 95% CI 0.86-1.00) and OS (HR 0.89, 95% CI 0.81-0.98).

Genomic markers for tumor proliferation assessed were *AURKA*, *MYBL2*, and *MKI67*. *AURKA* (N = 79) demonstrated a significant prognostic impact on DFS (HR 1.03, 95% CI 1.01-1.04) and OS (HR 1.02, 95% CI 1.01-1.04). *MYBL2* expression (N = 79) also correlated with lower DFS (HR 1.10, 95% CI 1.02-1.19) and OS (HR 1.10, 95% CI 1.03-1.18). The proliferation marker *MKI67* (N = 79) only exhibited prognostic significance for OS (HR 1.07, 95% CI 1.00-1.14).

Clinically relevant variables and biomarkers that had previously exhibited prognostic significance were included to generate multivariate Cox models. *MKI67*, that showed significant correlation with *MYBL2*, was excluded to prevent collinearity (**Supplementary Table 1**). Thus, together with HR/HER2 status and response to NCT, the NLR, TILs, and *AURKA* and *MYBL2* expression remained as independent prognostic variables for both DFS and OS (**Table 2**).

**Table 2:** Multivariate analysis of immune response-related and proliferation markers for disease- free survival and overall survival.

Variables	DFS			OS		
	HR	95% CI	P	HR	95% CI	P
pCR	0.01	0.01 – 0.78	0.042	0.04	0.01 – 1.61	0.090
HR+	0.23	0.04 – 1.16	0.075	0.14	0.03 – 0.81	0.027
HER2+	0.15	0.14 – 1.74	0.131	0.25	0.03 – 2.10	0.204
NLR	1.80	1.12 – 2.88	0.014	1.37	1.03 – 1.83	0.033
TILs*	0.93	0.83 – 1.05	0.259	0.86	0.75 – 0.98	0.026
AURKA*	1.04	1.00 – 1.08	0.069	1.04	1.00 – 1.09	0.090
MYBL2*	1.13	1.01 – 1.27	0.037	1.19	1.05 – 1.35	0.007

AURKA, Aurora kinase A; DFS, disease-free survival; HER2+, human epidermal growth factor receptor 2 positive; HR, hazard ratio; HR+, hormone receptors positive; MYBL2, MYB Proto-Oncogene Like 2; NLR, neutrophil-to-lymphocyte ratio; OS, overall survival; pCR, pathological complete response; TILs, tumor-infiltrating lymphocytes; 95% CI, 95% confidence interval.

*Square root transformation of these variables was performed.

### 3.3 Analysis of the prognostic capacity of the different models

**Figure 2:**
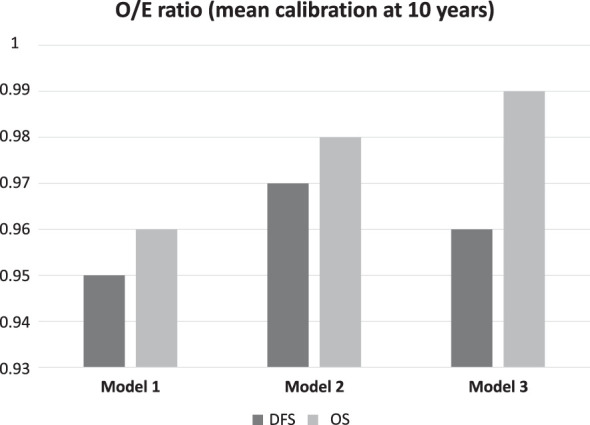
Mean calibration at 10 years. O/E (observed/expected) ratios for each model are shown, both for OS and DFS.

We assessed the predictive performance of the biomarkers included in the multivariate Cox proportional hazards models using AIC and LLRT. We consecutively added the immune response biomarkers, NLR and TILs (model 2), and genomic markers, *AURKA* and *MYBL2* (model 3), to HR/HER2 status and NCT response (model 1). For both DFS and OS, we established that the consecutive addition of biomarkers generated a progressive increase in the models’ predictive capacity (**Table 3**). This was reflected in a gradual decrease in AIC for both DFS (AIC model 3: 64 vs AIC model 1: 79) and OS (AIC model 3: 58 vs AIC model 1: 70), in addition to differences in LLRT for the different models that were statistically significant compared to the isolated clinical parameter (DFS: p < 0.001; OS: p = 0.005). A side-by-side comparison between this combinatorial model versus prediction using individual markers, is provided as **Supplementary Table 2**. Additionally, the 10 years mean calibration of the OS model also improved with the addition of proliferation and immune markers, while it remained virtually unchanged for the DFS models (**Figure 2** and **Supplementary Table 3**).

**Table 3:** Predictive capacity following the consecutive addition of biomarkers to clinical variables.

Models	DFS					OS				
	AIC	LLR†	P†	LLR‡	P‡	AIC	LLR†	P†	LLR‡	P‡
Model 1 pCR + HR/HER2 status	79	22.50	<0.001	15.46	<0.001	70	20.69	<0.001	10.71	0.005
Model 2 + NLR + TILs	68	7.04	0.030	ref	n/a	64	9.98	0.007	ref	n/a
Model 3 +*AURKA* + *MYBL2*	64	ref	n/a	n/a	n/a	58	ref	n/a	n/a	n/a

†Likelihood ratio test (LLRT) and p value for comparison of model 3 (reference category) and models 1 and 2 (nested models).

‡ Likelihood ratio test (LLRT) and p value for comparison of model 2 (reference category) and model 1 (nested model).

AIC, Akaike Information Criterion; AURKA, Aurora kinase A; DFS, disease-free survival; HER2, human epidermal growth factor receptor 2; HR, hormone receptor; NLR, neutrophil-to-lymphocyte ratio; MYBL2, MYB Proto-Oncogene Like 2; OS, overall survival; pCR, pathological complete response; TILs, tumor-infiltrating lymphocytes; Ref, reference; N/a, not applicable.

**Figure 3:**
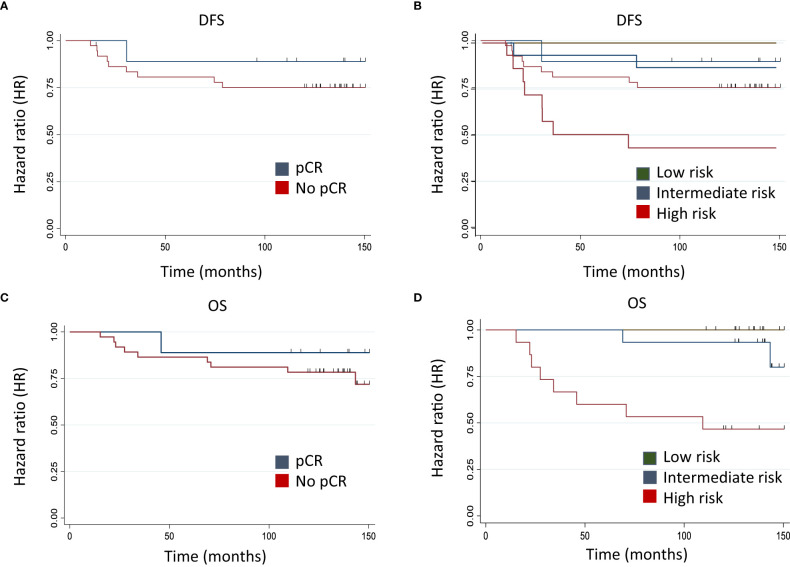
Model prediction with Kaplan-Meier curves for DFS and OS. Kaplan-Meier curves for DFS and OS obtained with predictions from clinical variable (pCR) **(A, C)** and model 3 (pCR, NLR, TILs *AURKA*, and *MYBL2*) **(B, D)**, the latter divided into tertiles. Model 1: PLR DFS: 0.34 - PLR OS: 0.41. Model 3: PLR DFS: 0.0014 - PLR OS: 0.0054.

The predictive value added by the biomarkers was also gauged by plotting cohort survival as a function of the predictions obtained with each model. **Figure 3** depicts the Kaplan-Meier curves for DFS and OS obtained with the predictions of pCR and model 3 (pCR, HR and HER2 status, NLR, TILs, *AURKA*, and *MYBL2*), the latter divided into tertiles. This exploratory analysis showed that the incorporation of the immunological and proliferation parameters with the clinical variable (pCR) in model 3 allowed a better stratification of the risk of events in the cohort analyzed than model 1. In fact, while the log-rank test indicated no statistically significant differences for model 1, such differences were statistically significant for model 3, both for DFS (p = 0.0014) and OS (p = 0.0054).

## 4 Discussion

The expanding body of knowledge of the immune response to BC and the differences between the systemic and microenvironment response provides opportunities for identifying predictive and prognostic biomarkers (10, 42). In the challenging context of neoadjuvant BC treatment, it is likely that several biomarkers will need to be combined to improve prognostic stratification (39, 43). Although pCR has been considered a surrogate marker of survival in early BC patients treated with NCT, in the luminal subtype we should probably consider additional markers to achieve better survival prediction. Moreover, the selection of patients who may gain real benefit from chemotherapy and those who may not is a pressing issue in the investigation of new therapeutic strategies and the avoidance of unnecessary chemotherapy side effects.

To explore new combination prognostic biomarkers, we conducted this study in a cohort of NCT-treated BC patients and investigated the prognostic implications for both DFS and OS of immune biomarkers in peripheral blood, such as NLR, and at the tumor level (TILs), together with genomic proliferation markers (*AURKA*, *MYBL2*, and *MKI67*) and conventional parameters, and response to NCT. Integrating all of these factors in a prognostic model consisting of NLR, TIL, *AURKA* and *MYBL2*, as a complement to HR/HER2 status and response to NCT, displayed a remarkable capacity to predict relapse and death.

Individually, NLR and TILs reflect systemic and local immune status, respectively. Specifically, for NLR, the results we obtained in our cohort were consistent with most of the literature reported to date. Zhoe et al. confirmed in a recent meta-analysis with 5504 BC patients treated with NCT that an NLR < 2.3 was predictive of pCR independently of tumor stage or grade and KI67 expression level (44). They also identified NLR as a prognostic biomarker, with patients with higher NLR levels having worse DFS (44). A previous meta-analysis of 8563 patients reached similar conclusions, with a large NLR cut-off range (1.9 – 5) and a median of 3 (14). In our study, we evaluated NLR as a continuous quantitative variable, thus avoiding the selection of an arbitrary or data-driven cut-off point. To better understand the significance of the NLR, we should probably investigate the dynamic change of NLR associated with chemotherapy. It has been reported that a lower NLR after chemotherapy predicts better pCR (45), while another study demonstrated that this change could be a predictor of pCR beyond the third NCT cycle (46). This is consistent with previous data generated by our group, that evaluated the prognostic value of peripheral blood lymphocytes and changes associated with NCT in BC patients (47).

The prognostic implications of TILs were also in line with earlier works (7, 48). The 12-year follow-up of our series of BC patients who received NCT is one of the longest published. It is relevant that in this 12-year study, TILs predict the same good prognosis as we reported in our 5-year study (9). One strength of this analysis is that, since there is no valid cut-off point and the evidence available is extremely heterogeneous, we have evaluated TILs as a continuous quantitative variable, which is the recommended approach in these cases (49). The positive response and outcome of women with greater lymphocyte infiltration could be, at least in part, due to the activation of the antitumor immune response during NCT, induced after DNA damage and cell death (50). A meta-analysis of 18170 BC patients confirmed high TILs as a predictive and prognostic biomarker in HER2+ and TNBC. In luminal subtypes, high TILs were correlated with poor prognosis (51). Unfortunately, our series is small for BC subtype analyses. Luminal BC is characterized by low TIL infiltration with low HLA expression, so other immune cells such as tumor associated macrophages (TAM) could be more relevant than T cells (52) or even NK cells whose role has not yet been studied in depth in BC. In this line, it is essential to determine the infiltrating lymphocyte subtypes in BC (9, 53). The significance of defining the immune-infiltrating cell type has been studied by an immune risk score analyses using TGCA and other database genes. A low prognostic immune risk score with five cell subtypes (B cells, endothelial cells, macrophages, NK cells, other cells) has been correlated with better survival in BC (54).

The correlation between NLR and TILs and their combined predictive capacity for adverse events has yet to be fully elucidated. Our study confirms the lack of correlation between both variables, suggesting that systemic and local immunities respond to different regulatory mechanisms (55); therefore, combined predictive models can offer a more comprehensive vision (39).This lack of correlation is consistent with previously published findings in TNBC and luminal BC (55–58). In our cohort, not only did both remain as independent prognostic variables in the multivariate analysis, but the combination of NLR and TILs improved the prognostic accuracy over pCR to NCT alone.

Genomics has improved our understanding of BC biology and revealed four intrinsic molecular subtypes with significant immunological differences, as previously mentioned (43). Immune-activated gene subsets, higher expression of proimmune factors and/or TILs have been associated with chemosensitivity, but also proliferation has been identified as a key factor in BC (59). Particularly, in the context of BC patients treated with NCT, proliferation markers correlate with oncologic progression and prognosis for the different IHC subtypes (60, 61). Therefore, the information they provide might complement what we learn from immunological biomarkers (61, 62). The choice of the optimal combination of proliferation markers was a difficult decision to make. Wirapati et al. published a meta-analysis of gene expression profiles with three gene modules (proliferation, ER signaling and *ERBB2* amplification) in 2833 breast tumor to better understand cancer subtyping and prognosis signatures (63). *AURKA* was chosen as a proliferation gene based on this study and others already discussed in the introduction (23, 24, 64). As we mentioned previously, *MYBL2* is one of the proliferation genes included in Oncotype, PAM50 and MammaPrint tests (19). Finally, *MKI67* was chosen since Ki67 is used in the clinical setting to identify luminal B cases and the correlation between Ki67 and *MKI67* has been previously established (65). One interesting finding is the statistically significant association observed between genomic markers *MKI67* and *MYBL2*. Previous reports have shown that the expression of MKI67 can be regulated by MYBL2, and this regulation is not specific to BC (66, 67). In our cohort, we did not observe a significant correlation between the proliferation markers *AURKA* and *MYBL2*, although previous work have noted a shared transcription between them (68).

Our study has demonstrated the independent prognostic value of the combination of systemic and microenvironmental immune biomarkers (NLR and TILs) with genomic proliferation markers (*AURKA* and *MYBL2*) and classical clinical factors (HR/HER status, pCR). Compellingly, the addition of proliferation biomarkers markedly increased the discriminatory capacity of the model. The model combining all variables (HR/HER status, pCR, NLR, TILs, *AURKA*, and *MYBL2*) exhibited the greatest predictive ability for DFS and OS, both in terms of AIC and statistically significance (LLRT). To date, the factors associated with response to NCT have been obtained from clinical, pathological and molecular analyses. However, the majority of these studies include a limited sample size, combine data from patients with different therapeutic strategies and use profiles with isolated variables that fail to capture the complexity of the tumor ecosystem. As a result, empirical clinical risk stratification continues to be used for selecting patients who are candidates for neoadjuvant treatment (69).

Several studies have explored the combination of local immune biomarkers associated with classical clinical factors such as response to neoadjuvant treatment (39). In particular, TILs, PD-L1 and genomic signatures and their combinations have been extensively studied (39, 69). Prat et al. compared the three-gene model (SCMGENE) with *ESR1*, *ERBB2* and *AURKA* with that of the PAM50 test. They concluded that while both models were able to anticipate patient outcomes, PAM50 was the superior model and the only one with predictive value. This was because PAM50 reveals biological diversity better than the three-gene model (70). Interestingly, our results are consistent with the results obtained using the three-gene model.

In this context, it is important to remark the potential benefit of a model that combines multiple biomarkers, in which all variable contribute to provide comprehensive patient information. Such a novel approach can provide an integrated immuno-genetic-oncological biomarker for selecting the most accurate therapy strategy. Furthermore, the available evidence suggest the potential value of the systematic implementation of combined biomarkers to improve patient selection and safety (39). To identify the most appropriate combined model, it is necessary to start exploring some of the most extensively studied biomarkers, as we have done in our original research.

Our study has some limitations. First, the method used to quantify TILs was based on IHC for CD3, which includes both stromal and intra-tumor lymphocytic infiltration and differs from the currently accepted method (49). However, our group has previously shown good correlation for TILs measured by IHC for CD3 with HE-based assessment of lymphocyte infiltration (9). Second, the use of genomic markers of proliferation differs from the more recent routine use of KI67 (71), although genomic signatures of proliferation are the strongest factor for prognostic stratification in most predictive genomic tests in early luminal BC (60, 72) and also comprise a key prognostic marker in non-luminal subtypes (73, 74). Finally, this is a small, single-center, retrospective cohort, in which it was not possible to perform subgroup analyses according to tumor subtype with sufficient statistical power to draw conclusions.

Nonetheless, our series does have a number of strengths that make it compelling. Besides having one of the longest follow-up published in literature, it is highly homogeneous with respect to clinical management, including diagnosis, NCT, and response determination, which are key to obtain and interpret data within the context of early BC. NCT, surgery and radiation therapy were performed according to usual clinical procedures and following current clinical practice guidelines, which makes treatment bias unlikely for the final prognostic model. Furthermore, the inclusion in our model of clinical classical factors, local immunological factors, such as TILs, systemic immune status, such as NLR, and two of the most relevant proliferation markers, is a novel approach in the study of breast cancer prognosis. These results warrant prospective, multi-centre validation studies with a larger sample size.

## 5 Conclusions

In conclusion, our study reveals that combining systemic immune and proliferation biomarkers with clinical-pathological markers improved the predictive capacity for DFS and OS compared to treatment response alone in a cohort of BC patients treated with NCT. The real benefit and clinical usefulness of these biomarker-based models should be confirmed in broader series. The validation of these findings in independent cohorts could provide a new tool for improving prognostic stratification and therapeutic management in these patients.

## Data availability statement

The data that support the findings of this study are available from the corresponding author upon reasonable request.

## Ethics statement

The studies involving human participants were reviewed and approved by Clinical Research and Trials Comittee of the University Hospital Morales Meseguer. The patients/participants provided their written informed consent to participate in this study.

## Author contributions

Concept, EG-T, EM, FP and EG-M. Data curation, EG-T, EM, EG-G, FP and EG-M. Formal analysis, EG-T, EM, FP and EG-M. Funding acquisition, FP. and EG-M. Investigation, EG-T, EM, GL-G, PB, MP-R, FP, and EG-M. Methodology, EG-T, EM, GL-G, MP-R, FP, and EG-M. Project administration, EG-T, EM, GL-G, FP and EG-M. Supervision, FP and EG-M. Writing—original draft, EG-T, FP and EG-M. Writing—review and editing, EG-T. EM, GL-G, PB., AB, MP-R, BA-A, AR, EG-G, FP, and EG-M. All authors contributed to the article and approved the submitted version.

## References

[1] Untch M Konecny GE Paepke S von Minckwitz G. Current and future role of neoadjuvant therapy for breast cancer. Breast (2014) 23:526–37. doi: 10.1016/j.breast.2014.06.004 25034931

[2] van Nes JGH Putter H Julien J-P Tubiana-Hulin M van de Vijver M Bogaerts J. Preoperative chemotherapy is safe in early breast cancer, even after 10 years of follow-up; clinical and translational results from the EORTC trial 10902. Breast Cancer Res Treat (2009) 115:101–13. doi: 10.1007/s10549-008-0050-1 18484198

[3] Haque W Verma V Hatch S Suzanne Klimberg V Brian Butler E Teh BS. Response rates and pathologic complete response by breast cancer molecular subtype following neoadjuvant chemotherapy. Breast Cancer Res Treat (2018) 170:559–67. doi: 10.1007/s10549-018-4801-3 29693228

[4] Cortazar P Zhang L Untch M Mehta K Costantino JP Wolmark N. Pathological complete response and long-term clinical benefit in breast cancer: the CTNeoBC pooled analysis. Lancet (2014) 384:164–72. doi: 10.1016/S0140-6736(13)62422-8 24529560

[5] Mamounas EP Anderson SJ Dignam JJ Bear HD Julian TB Geyer CEJ. Predictors of locoregional recurrence after neoadjuvant chemotherapy: results from combined analysis of national surgical adjuvant breast and bowel project b-18 and b-27. J Clin Oncol (2012) 30:3960–6. doi: 10.1200/JCO.2011.40.8369 PMC3488269 23032615

[6] LeVasseur N Sun J Gondara L Diocee R Speers C Lohrisch C. Impact of pathologic complete response on survival after neoadjuvant chemotherapy in early-stage breast cancer: a population-based analysis. J Cancer Res Clin Oncol (2020) 146:529–36. doi: 10.1007/s00432-019-03083-y PMC11804444 31741041

[7] Denkert C von Minckwitz G Darb-Esfahani S Lederer B Heppner BI Weber KE. Tumour-infiltrating lymphocytes and prognosis in different subtypes of breast cancer: a pooled analysis of 3771 patients treated with neoadjuvant therapy. Lancet Oncol (2018) 19:40–50. doi: 10.1016/S1470-2045(17)30904-X 29233559

[8] Dieci MV Radosevic-Robin N Fineberg S van den Eynden G Ternes N Penault-Llorca F. Update on tumor-infiltrating lymphocytes (TILs) in breast cancer, including recommendations to assess TILs in residual disease after neoadjuvant therapy and in carcinoma in situ: a report of the international immuno-oncology biomarker working group on breast cancer. Semin Cancer Biol (2018) 52:16–25. doi: 10.1016/j.semcancer.2017.10.003 29024776

[9] García-Martínez E Gil GL Benito AC González-Billalabeitia E Conesa MAV García García T. Tumor-infiltrating immune cell profiles and their change after neoadjuvant chemotherapy predict response and prognosis of breast cancer. Breast Cancer Res (2014) 16:488. doi: 10.1186/s13058-014-0488-5 25432519 PMC4303200

[10] Hiam-Galvez KJ Allen BM Spitzer MH. Systemic immunity in cancer. Nat Rev Cancer (2021) 21:345–59. doi: 10.1038/s41568-021-00347-z PMC8034277 33837297

[11] Templeton AJ McNamara MG Šeruga B Vera-Badillo FE Aneja P Ocaña A. Prognostic role of neutrophil-to-lymphocyte ratio in solid tumors: a systematic review and meta-analysis. J Natl Cancer Inst (2014) 106:dju124. doi: 10.1093/jnci/dju124 24875653

[12] Cupp MA Cariolou M Tzoulaki I Aune D Evangelou E Berlanga-Taylor AJ. Neutrophil to lymphocyte ratio and cancer prognosis: an umbrella review of systematic reviews and meta-analyses of observational studies. BMC Med (2020) 18:360. doi: 10.1186/s12916-020-01817-1 33213430 PMC7678319

[13] McAndrew NP Bottalico L Mesaros C Blair IA Tsao PY Rosado JM. Effects of systemic inflammation on relapse in early breast cancer. NPJ Breast Cancer (2021) 7:7. doi: 10.1038/s41523-020-00212-6 33483516 PMC7822844

[14] Ethier J-L Desautels D Templeton A Shah PS Amir E. Prognostic role of neutrophil-to-lymphocyte ratio in breast cancer: a systematic review and meta-analysis. Breast Cancer Res (2017) 19:2. doi: 10.1186/s13058-016-0794-1 28057046 PMC5217326

[15] Ivars Rubio A Yufera JC de la Morena P Fernández Sánchez A Navarro Manzano E García Garre E. Neutrophil-lymphocyte ratio in metastatic breast cancer is not an independent predictor of survival, but depends on other variables. Sci Rep (2019) 9:16979. doi: 10.1038/s41598-019-53606-3 31740715 PMC6861311

[16] Cullinane C Creavin B O’Leary DP O’Sullivan MJ Kelly L Redmond HP. Can the neutrophil to lymphocyte ratio predict complete pathologic response to neoadjuvant breast cancer treatment? a systematic review and meta-analysis. Clin Breast Cancer (2020) 20:e675–81. doi: 10.1016/j.clbc.2020.05.008 32653471

[17] Yerushalmi R Woods R Ravdin PM Hayes MM Gelmon KA. Ki67 in breast cancer: prognostic and predictive potential. Lancet Oncol (2010) 11:174–83. doi: 10.1016/S1470-2045(09)70262-1 20152769

[18] Paik S Shak S Tang G Kim C Baker J Cronin M. A multigene assay to predict recurrence of tamoxifen-treated, node-negative breast cancer. N Engl J Med (2004) 351:2817–26. doi: 10.1056/NEJMoa041588 15591335

[19] Beňačka R Szabóová D Guľašová Z Hertelyová Z Radoňák J. Classic and new markers in diagnostics and classification of breast cancer. Cancers (Basel) (2022) 14(21):5444–5466. doi: 10.3390/cancers14215444 36358862 PMC9654192

[20] Kalinsky K Barlow WE Gralow JR Meric-Bernstam F Albain KS Hayes DF. 21-gene assay to inform chemotherapy benefit in node-positive breast cancer. N Engl J Med (2021) 385:2336–47. doi: 10.1056/NEJMoa2108873 PMC9096864 34914339

[21] Du R Huang C Liu K Li X Dong Z. Targeting AURKA in cancer: molecular mechanisms and opportunities for cancer therapy. Mol Cancer (2021) 20:15. doi: 10.1186/s12943-020-01305-3 33451333 PMC7809767

[22] Whately KM Voronkova MA Maskey A Gandhi J Loskutov J Choi H. Nuclear aurora-a kinase-induced hypoxia signaling drives early dissemination and metastasis in breast cancer: implications for detection of metastatic tumors. Oncogene (2021) 40:5651–64. doi: 10.1038/s41388-021-01969-1 PMC9511212 34326467

[23] Xu J Wu X Zhou W Liu A Wu J Deng J. Aurora-a identifies early recurrence and poor prognosis and promises a potential therapeutic target in triple negative breast cancer. PLoS One (2013) 8:e56919. doi: 10.1371/journal.pone.0056919 23437271 PMC3577665

[24] Siggelkow W Boehm D Gebhard S Battista M Sicking I Lebrecht A. Expression of aurora kinase a is associated with metastasis-free survival in node-negative breast cancer patients. BMC Cancer (2012) 12:562. doi: 10.1186/1471-2407-12-562 23186136 PMC3530429

[25] Jalalirad M Haddad TC Salisbury JL Radisky D Zhang M Schroeder M. Aurora-a kinase oncogenic signaling mediates TGF-β-induced triple-negative breast cancer plasticity and chemoresistance. Oncogene (2021) 40:2509–23. doi: 10.1038/s41388-021-01711-x PMC8032554 33674749

[26] Zhang Y Wang Y Xue J. Paclitaxel inhibits breast cancer metastasis via suppression of aurora kinase-mediated cofilin-1 activity. Exp Ther Med (2018) 15:1269–76. doi: 10.3892/etm.2017.5588 PMC5776659 29434713

[27] Zhou T Zhang A Kuang G Gong X Jiang R Lin D. Baicalin inhibits the metastasis of highly aggressive breast cancer cells by reversing epithelial-to-mesenchymal transition by targeting β-catenin signaling. Oncol Rep (2017) 38:3599–607. doi: 10.3892/or.2017.6011 29039569

[28] Hole S Pedersen AM Lykkesfeldt AE Yde CW. Aurora kinase a and b as new treatment targets in aromatase inhibitor-resistant breast cancer cells. Breast Cancer Res Treat (2015) 149:715–26. doi: 10.1007/s10549-015-3284-8 25667100

[29] Lykkesfeldt AE Iversen BR Jensen M-B Ejlertsen B Giobbie-Hurder A Reiter BE. Aurora kinase a as a possible marker for endocrine resistance in early estrogen receptor positive breast cancer. Acta Oncol (2018) 57:67–73. doi: 10.1080/0284186X.2017.1404126 29202611

[30] Donnella HJ Webber JT Levin RS Camarda R Momcilovic O Bayani N. Kinome rewiring reveals AURKA limits PI3K-pathway inhibitor efficacy in breast cancer. Nat Chem Biol (2018) 14:768–77. doi: 10.1038/s41589-018-0081-9 PMC6051919 29942081

[31] Wander SA Cohen O Gong X Johnson GN Buendia-Buendia JE Lloyd MR. The genomic landscape of intrinsic and acquired resistance to cyclin-dependent kinase 4/6 inhibitors in patients with hormone receptor-positive metastatic breast cancer. Cancer Discovery (2020) 10:1174–93. doi: 10.1158/2159-8290.CD-19-1390 PMC8815415 32404308

[32] Morris BB Smith JP Zhang Q Jiang Z Hampton OA Churchman ML. Replicative instability drives cancer progression. Biomolecules (2022) 12(11):1570–1592. doi: 10.3390/biom12111570 36358918 PMC9688014

[33] Shi H Bevier M Johansson R Enquist-Olsson K Henriksson R Hemminki K. Prognostic impact of polymorphisms in the MYBL2 interacting genes in breast cancer. Breast Cancer Res Treat (2012) 131:1039–47. doi: 10.1007/s10549-011-1826-2 22037783

[34] Bayley R Ward C Garcia P. MYBL2 amplification in breast cancer: molecular mechanisms and therapeutic potential. Biochim Biophys Acta Rev Cancer (2020) 1874:188407. doi: 10.1016/j.bbcan.2020.188407 32853735

[35] Tao D Pan Y Jiang G Lu H Zheng S Lin H. B-myb regulates snail expression to promote epithelial-to-mesenchymal transition and invasion of breast cancer cell. Med Oncol (2015) 32:412. doi: 10.1007/s12032-014-0412-y 25502082

[36] Chen X Lu Y Yu H Du K Zhang Y Nan Y. Pan-cancer analysis indicates that MYBL2 is associated with the prognosis and immunotherapy of multiple cancers as an oncogene. Cell Cycle (2021) 20:2291–308. doi: 10.1080/15384101.2021.1982494 PMC8794527 34585645

[37] Li X Zhang X Wu C-C Li P-P Fu Y-M Xie L-H. The role of MYB proto-oncogene like 2 in tamoxifen resistance in breast cancer. J Mol Histol (2021) 52:21–30. doi: 10.1007/s10735-020-09920-6 33141360

[38] Guarneri V Dieci MV Bisagni G Brandes AA Frassoldati A Cavanna L. PIK3CA mutation in the ShortHER randomized adjuvant trial for patients with early HER2(+) breast cancer: association with prognosis and integration with PAM50 subtype. Clin Cancer Res (2020) 26:5843–51. doi: 10.1158/1078-0432.CCR-20-1731 32843527

[39] Gonzalez-Ericsson PI Stovgaard ES Sua LF Reisenbichler E Kos Z Carter JM. The path to a better biomarker: application of a risk management framework for the implementation of PD-L1 and TILs as immuno-oncology biomarkers in breast cancer clinical trials and daily practice. J Pathol (2020) 250:667–84. doi: 10.1002/path.5406 32129476

[40] Filho OM Stover DG Asad S Ansell PJ Watson M Loibl S. Association of immunophenotype with pathologic complete response to neoadjuvant chemotherapy for triple-negative breast cancer: a secondary analysis of the BrighTNess phase 3 randomized clinical trial. JAMA Oncol (2021) 7:603–8. doi: 10.1001/jamaoncol.2020.7310 PMC7893540 33599688

[41] Carlson RW Anderson BO Burstein HJ Carter WB Edge SB Farrar WB. Invasive breast cancer. J Natl Compr Canc Netw (2007) 5:246–312. doi: 10.6004/jnccn.2007.0025 17439758

[42] Dieci MV Miglietta F Guarneri V. Immune infiltrates in breast cancer: recent updates and clinical implications. Cells (2021) 10(2):223–250. doi: 10.3390/cells10020223 33498711 PMC7911608

[43] Hammerl D Smid M Timmermans AM Sleijfer S Martens JWM Debets R. Breast cancer genomics and immuno-oncological markers to guide immune therapies. Semin Cancer Biol (2018) 52:178–88. doi: 10.1016/j.semcancer.2017.11.003 29104025

[44] Zhou Q Dong J Sun Q Lu N Pan Y Han X. Role of neutrophil-to-lymphocyte ratio as a prognostic biomarker in patients with breast cancer receiving neoadjuvant chemotherapy: a meta-analysis. BMJ Open (2021) 11:e047957. doi: 10.1136/bmjopen-2020-047957 PMC8475153 34561257

[45] Dan J Tan J Huang J Zhang X Guo Y Huang Y. The dynamic change of neutrophil to lymphocyte ratio is predictive of pathological complete response after neoadjuvant chemotherapy in breast cancer patients. Breast Cancer (2020) 27:982–8. doi: 10.1007/s12282-020-01096-x 32306184

[46] Choi H Noh H Cho I-J Lim S-T Han A. Changes in neutrophil to lymphocyte ratio (NLR) during neoadjuvant treatment correlated with patients’ survival. Breast Cancer (2020) 27:871–9. doi: 10.1007/s12282-020-01083-2 32221862

[47] Vicente Conesa MA Garcia-Martinez E Gonzalez Billalabeitia E Chaves Benito A Garcia Garcia T Vicente Garcia V. Predictive value of peripheral blood lymphocyte count in breast cancer patients treated with primary chemotherapy. Breast (2012) 21:468–74. doi: 10.1016/j.breast.2011.11.002 22119767

[48] Denkert C Loibl S Noske A Roller M Müller BM Komor M. Tumor-associated lymphocytes as an independent predictor of response to neoadjuvant chemotherapy in breast cancer. J Clin Oncol (2010) 28:105–13. doi: 10.1200/JCO.2009.23.7370 19917869

[49] Salgado R Denkert C Demaria S Sirtaine N Klauschen F Pruneri G. The evaluation of tumor-infiltrating lymphocytes (TILs) in breast cancer: recommendations by an international TILs working group 2014. Ann Oncol (2015) 26:259–71. doi: 10.1093/annonc/mdu450 PMC6267863 25214542

[50] Chen DS Mellman I. Oncology meets immunology: the cancer-immunity cycle. Immunity (2013) 39:1–10. doi: 10.1016/j.immuni.2013.07.012 23890059

[51] Gao Z-H Li C-X Liu M Jiang J-Y. Predictive and prognostic role of tumour-infiltrating lymphocytes in breast cancer patients with different molecular subtypes: a meta-analysis. BMC Cancer (2020) 20:1150. doi: 10.1186/s12885-020-07654-y 33238978 PMC7690150

[52] Goldberg J Pastorello RG Vallius T Davis J Cui YX Agudo J. The immunology of hormone receptor positive breast cancer. Front Immunol (2021) 12:674192. doi: 10.3389/fimmu.2021.674192 34135901 PMC8202289

[53] Mao P Cohen O Kowalski KJ Kusiel JG Buendia-Buendia JE Cuoco MS. Acquired FGFR and FGF alterations confer resistance to estrogen receptor (ER) targeted therapy in ER(+) metastatic breast cancer. Clin Cancer Res (2020) 26:5974–89. doi: 10.1158/1078-0432.CCR-19-3958 32723837

[54] Sun Y Zhang C. The types of tumor infiltrating lymphocytes are valuable for the diagnosis and prognosis of breast cancer. Front Genet (2022) 13:1019062. doi: 10.3389/fgene.2022.1019062 36386851 PMC9641369

[55] Pang J Zhou H Dong X Wang S Xiao Z. Relationship between the neutrophil to lymphocyte ratio, stromal tumor-infiltrating lymphocytes, and the prognosis and response to neoadjuvant chemotherapy in triple-negative breast cancer. Clin Breast Cancer (2021) 21:e681–7. doi: 10.1016/j.clbc.2021.04.004 34001439

[56] Dong X Liu C Yuan J Wang S Ding N Li Y. Prognostic roles of neutrophil-to-Lymphocyte ratio and stromal tumor-infiltrating lymphocytes and their relationship in locally advanced triple-negative breast cancer treated with neoadjuvant chemotherapy. Breast Care (Basel) (2021) 16:328–34. doi: 10.1159/000509498 PMC8436630 34602938

[57] Lee J Kim D-M Lee A. Prognostic role and clinical association of tumor-infiltrating lymphocyte, programmed death ligand-1 expression with neutrophil-lymphocyte ratio in locally advanced triple-negative breast cancer. Cancer Res Treat (2019) 51:649–63. doi: 10.4143/crt.2018.270 PMC6473269 30064200

[58] Bun A Fujimoto Y Higuchi T Sata A Fukui R Ozawa H. Prognostic significance of neutrophil-to-lymphocyte ratio in luminal breast cancers with low levels of tumour-infiltrating lymphocytes. Anticancer Res (2020) 40:2871–80. doi: 10.21873/anticanres.14263 32366437

[59] Denkert C Loibl S Müller BM Eidtmann H Schmitt WD Eiermann W. Ki67 levels as predictive and prognostic parameters in pretherapeutic breast cancer core biopsies: a translational investigation in the neoadjuvant GeparTrio trial. Ann Oncol (2013) 24:2786–93. doi: 10.1093/annonc/mdt350 23970015

[60] Torrisi R Marrazzo E Agostinetto E De Sanctis R Losurdo A Masci G. Neoadjuvant chemotherapy in hormone receptor-positive/HER2-negative early breast cancer: when, why and what? Crit Rev Oncol Hematol (2021) 160:103280. doi: 10.1016/j.critrevonc.2021.103280 33667658

[61] Sinn BV Loibl S Hanusch CA Zahm D-M Sinn H-P Untch M. Immune-related gene expression predicts response to neoadjuvant chemotherapy but not additional benefit from PD-L1 inhibition in women with early triple-negative breast cancer. Clin Cancer Res (2021) 27:2584–91. doi: 10.1158/1078-0432.CCR-20-3113 33593886

[62] Wang K Li H-L Xiong Y-F Shi Y Li Z-Y Li J. Development and validation of nomograms integrating immune-related genomic signatures with clinicopathologic features to improve prognosis and predictive value of triple-negative breast cancer: a gene expression-based retrospective study. Cancer Med (2019) 8:686–700. doi: 10.1002/cam4.1880 30677255 PMC6382728

[63] Wirapati P Sotiriou C Kunkel S Farmer P Pradervand S Haibe-Kains B. Meta-analysis of gene expression profiles in breast cancer: toward a unified understanding of breast cancer subtyping and prognosis signatures. Breast Cancer Res (2008) 10:R65. doi: 10.1186/bcr2124 18662380 PMC2575538

[64] Fadaka AO Sibuyi NRS Madiehe AM Meyer M. MicroRNA-based regulation of aurora a kinase in breast cancer. Oncotarget (2020) 11:4306–24. doi: 10.18632/oncotarget.27811 PMC7679040 33245732

[65] Chen L Chen Y Xie Z Luo J Wang Y Zhou J. Comparison of immunohistochemistry and RT-qPCR for assessing ER, PR, HER2, and Ki67 and evaluating subtypes in patients with breast cancer. Breast Cancer Res Treat (2022) 194:517–29. doi: 10.1007/s10549-022-06649-6 35789315

[66] Qin H Li Y Zhang H Wang F He H Bai X. Prognostic implications and oncogenic roles of MYBL2 protein expression in esophageal squamous-cell carcinoma. Onco Targets Ther (2019) 12:1917–27. doi: 10.2147/OTT.S190145 PMC6415733 30881043

[67] Luengo-Gil G García-Martínez E Chaves-Benito A Conesa-Zamora P Navarro-Manzano E González-Billalabeitia E. Clinical and biological impact of miR-18a expression in breast cancer after neoadjuvant chemotherapy. Cell Oncol (Dordr) (2019) 42:627–44. doi: 10.1007/s13402-019-00450-2 PMC12994329 31115881

[68] Musa J Aynaud M-M Mirabeau O Delattre O Grünewald TG. MYBL2 (B-myb): a central regulator of cell proliferation, cell survival and differentiation involved in tumorigenesis. Cell Death Dis (2017) 8:e2895. doi: 10.1038/cddis.2017.244 28640249 PMC5520903

[69] Sammut S-J Crispin-Ortuzar M Chin S-F Provenzano E Bardwell HA Ma W. Multi-omic machine learning predictor of breast cancer therapy response. Nature (2022) 601:623–9. doi: 10.1038/s41586-021-04278-5 PMC8791834 34875674

[70] Prat A Parker JS Fan C Perou CM. PAM50 assay and the three-gene model for identifying the major and clinically relevant molecular subtypes of breast cancer. Breast Cancer Res Treat (2012) 135:301–6. doi: 10.1007/s10549-012-2143-0 PMC3413822 22752290

[71] Nielsen TO Leung SCY Rimm DL Dodson A Acs B Badve S. Assessment of Ki67 in breast cancer: updated recommendations from the international Ki67 in breast cancer working group. J Natl Cancer Inst (2021) 113:808–19. doi: 10.1093/jnci/djaa201 PMC8487652 33369635

[72] Buus R Sestak I Kronenwett R Ferree S Schnabel CA Baehner FL. Molecular drivers of oncotype DX, prosigna, EndoPredict, and the breast cancer index: a TransATAC study. J Clin Oncol (2021) 39:126–35. doi: 10.1200/JCO.20.00853 PMC8078458 33108242

[73] Stover DG Coloff JL Barry WT Brugge JS Winer EP Selfors LM. The role of proliferation in determining response to neoadjuvant chemotherapy in breast cancer: a gene expression-based meta-analysis. Clin Cancer Res (2016) 22:6039–50. doi: 10.1158/1078-0432.CCR-16-0471 PMC5161615 27330058

[74] Zhang Y Asad S Weber Z Tallman D Nock W Wyse M. Genomic features of rapid versus late relapse in triple negative breast cancer. BMC Cancer (2021) 21:568. doi: 10.1186/s12885-021-08320-7 34006255 PMC8130400

