# Supplementary material for: A new prognostic model including immune biomarkers, genomic proliferation tumor markers (AURKA and MYBL2) and clinical-pathological features optimizes prognosis in neoadjuvant breast cancer patients
Source: Front Oncol. 2023 May 29;13:1182725. doi: 10.3389/fonc.2023.1182725 (PMC10258327; doi:10.3389/fonc.2023.1182725)
Supplement: Supplementary file 1 [file DataSheet_1.docx]

Supplementary Material

A new prognostic model including immune biomarkers, genomic proliferation tumour markers (*AURKA* and *MYBL2*) and clinical-pathological features optimizes prognosis in neoadjuvant breast cancer patients

Esmeralda García-Torralba^1,2,3^, Esther Navarro Manzano^2,3^, Gines Luengo-Gil1^1,2,3^, Pilar De la Morena Barrio^1,2,3^, Asunción Chaves Benito^4^, Miguel Pérez-Ramos^4^, Beatriz Álvarez-Abril^1,2,3^, Alejandra Ivars Rubio^1,2,3^, Elisa García-Garre^1,2,3^, Francisco Ayala de la Peña^1,2,3*^ and Elena García-Martínez^1,2,3,5^

^1^ Department of Haematology and Medical Oncology, University Hospital Morales Meseguer, Murcia, Spain.

^2^ University of Murcia (UMU), Murcia, Spain.

^3^ IMIB, Murcia, Spain.

^4^ Department of Pathology, University Hospital Morales Meseguer, Murcia, Spain.

^5^ Catholic University of Murcia (UCAM), Murcia, Spain.

*** Correspondence:**

Francisco Ayala de la Peña (ORCID: 0000-0001-6311-920X).

Department of Haematology and Medical Oncology. University Hospital Morales Meseguer. Avda. Marqués de los Vélez, s/n. Murcia 30008, Spain.

# Email: frayala@um.es.

# Supplementary Figures and Tables

## Supplementary Figures


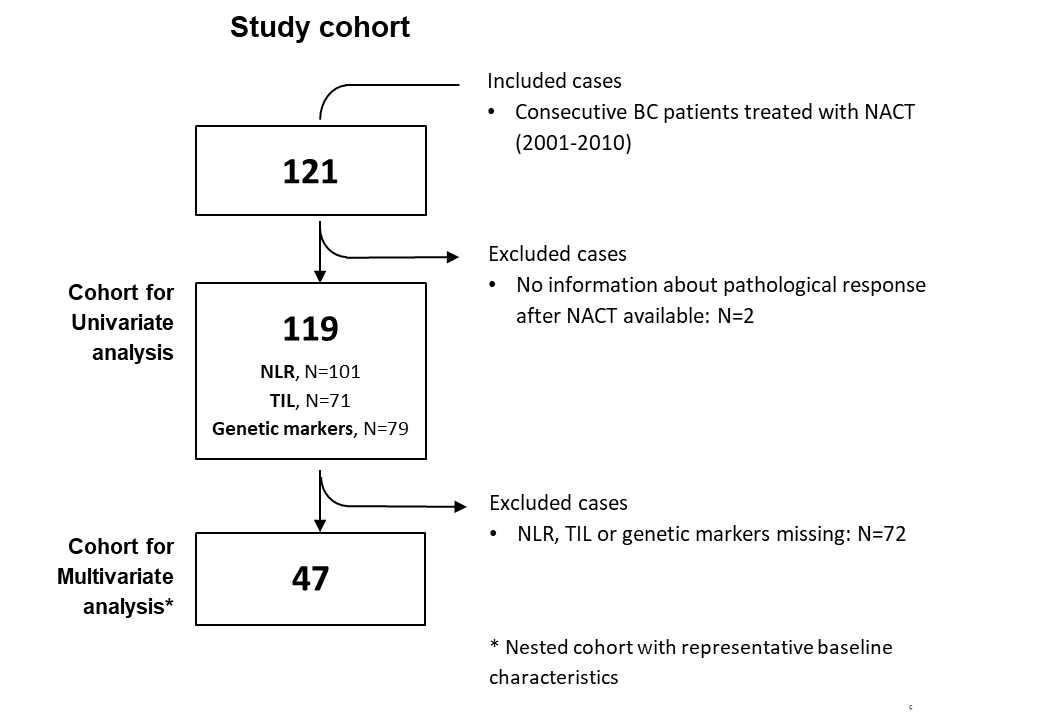


**Supplementary Figure 1. Flow chart.** *AURKA*: Aurora kinase A. BC: breast cancer. NLR: neutrophil-to-lymphocyte ratio. NCT: neoadjuvant chemotherapy. TILs: tumor infiltrating lymphocytes.

## Supplementary Tables

|  | | **NLR** | **TIL** | ***AURKA*** | ***MYBL2*** | ***MKI67*** |
| --- | --- | --- | --- | --- | --- | --- |
| **NLR** | Rho de Spearman | 1.000 |  |  |  |  |
| **TIL** | Rho de Spearman  p-value | 0.119  0.361 | 1.000 |  |  |  |
| ***AURKA*** | Rho de Spearman  p-value | 0.208  0.071 | 0.184  0.164 | 1.000 |  |  |
| ***MYBL2*** | Rho de Spearman  p-value | 0.236  0.052 | -0.004  0.977 | 0.124  0.284 | 1.000 |  |
| ***MKI67*** | Rho de Spearman  p-value | 0.076  0.534 | -0.053  0.701 | 0.077  0.507 | 0.856  0.000 | 1.000 |

**Supplementary table 1. Analysis of correlation across variables.** *AURKA*: Aurora kinase A. *MKI67*: Ki67 proliferation marker. MYBL2: MYB Proto-Oncogene Like 2. NLR: neutrophil-to-lymphocyte ratio. TILs: tumor infiltrating lymphocytes.

| **Variable** | **DFS** | | | **OS** | | |
| --- | --- | --- | --- | --- | --- | --- |
|  | **AIC** | **LLR†** | **P†** | **AIC** | **LLR†** | **P†** |
| **pCR** | 79 | 26.60 | <0.001 | 75 | 28.72 | <0.001 |
| **HR/HER2 status** | 80 | 25.67 | <0.001 | 72 | 24.43 | <0.001 |
| **NLR** | 75 | 22.52 | 0.001 | 71 | 24.83 | <0.001 |
| **TILs** | 77 | 24.02 | <0.001 | 68 | 22.59 | <0.001 |
| ***AURKA*** | 75 | 21.90 | 0.001 | 71 | 25.67 | <0.001 |
| ***MYBL2*** | 79 | 26.09 | <0.001 | 72 | 26.05 | <0.001 |
| **Model 3: pCR + HR/HER2 status**  **+ NLR + TILs +*AURKA* + *MYBL2*** | 64 | ref | n/a | 58 | ref | n/a |

**Supplementary Table 2. Predictive capacity of univariate models.**

†Likelihood ratio test (LLRT) and p value for comparison of model 3 (pCR, HR and HER2 status, NLR, TILs, AURKA, and MYBL2, reference category) and univariate models (nested models).

AIC: Akaike Information Criterion. *AURKA*: Aurora kinase A. DFS: disease-free survival. HER2: human epidermal growth factor receptor 2. HR: hormone receptor. NLR: neutrophil-to-lymphocyte ratio. *MYBL2*: MYB Proto-Oncogene Like 2. OS: overall survival. pCR: pathological complete response. TILs: tumour-infiltrating lymphocytes. Ref: reference. N/a: not applicable.

| **Models** | **DFS OS** | | | | |
| --- | --- | --- | --- | --- | --- |
|  | **O/E** | **95%CI** |  | **O/E** | **95%CI** |
| **Model 1**  **pCR + HR/HER2 status** | 0.95 | 0.51-1.77 |  | 0.96 | 0.50-1.84 |
| **Model 2**  **+ NLR + TILs** | 0.97 | 0.52-1.81 |  | 0.98 | 0.51-1.88 |
| **Model 3**  **+*AURKA* + *MYBL2*** | 0.96 | 0.51-1.78 |  | 0.99 | 0.52-1.91 |

**Supplementary Table 3. Ten-years mean calibration of the prognostic model following the consecutive addition of biomarkers to clinical variables.** O/E: observed/expected ratio at 10 years; 95% CI: 95% confidence interval.
